# Supplementary material for: Nuclear Retention of mRNAs Through Paraspeckle Protein Binding to a Sequence Determinant in 3′UTR
Source: Int J Mol Sci. 2025 Jul 5;26(13):6488. doi: 10.3390/ijms26136488 (PMC12250583; doi:10.3390/ijms26136488)
Supplement: Supplementary file 1 [file ijms-26-06488-s001.zip › ijms-3733104.pdf]

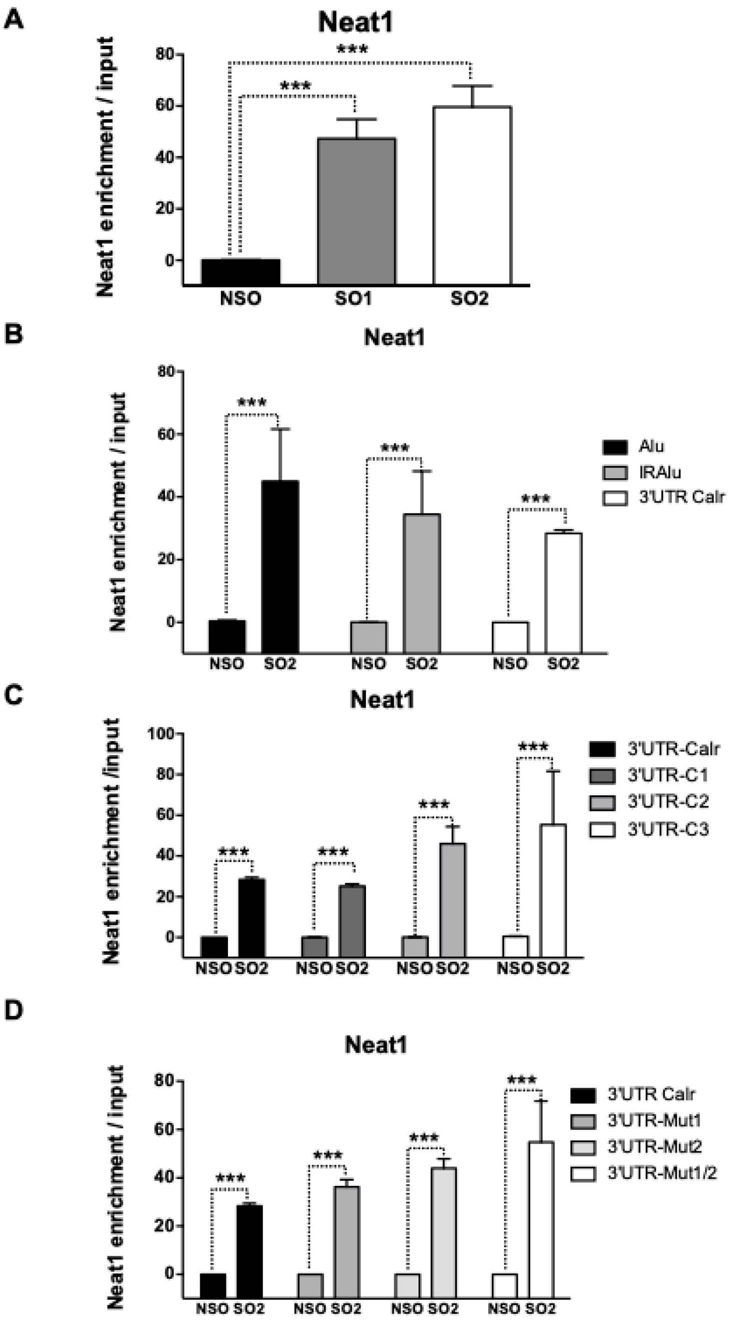

Supplemental Figure S1: Neat 1 RNA pull-down controls in the different experiments:

A. Enrichment of Neat1 RNA relative to input after RNA pull-down in native GH4C1 cells. Enrichment obtained after using the two anti-)àsens specific (Specific Oligonucleotide 1: SO1 and Specific Oligonucleotide 2 : SO2) or a non-specific (NSO) oligonucleotide probes. ***p<0.001vs non-specific oligonucleotide probe. **B.** Enrichment of Neat1 RNA relative to input after RNA pull-down in Alu-, IRAlu- and 3'UTR-Calr-containing Egfp mRNA cell lines. Enrichment obtained after using the anti-sens specific (Specific Oligonucleotide 2 : SO2) or a non-specific (NSO) oligonucleotide probes. ***p<0.001vs non-specific oligonucleotide probe. **C.** Enrichment of Neat1 RNA relative to input after RNA pull-down in 3'UTR-Calr-, 3'UTR-C1-, 3'UTR-C2- and 3'UTR-C3-containing Egfp mRNA cell lines. Enrichment obtained after using the anti-sens specific (Specific Oligonucleotide 2 : SO2) or a non-specific (NSO) oligonucleotide probes. ***p<0.001vs non-specific oligonucleotide

probe. D. Enrichment of Neat1 RNA relative to input after RNA pull-down in 3'UTR-Calr-, 3'UTR-Mut1-, 3'UTR-Mut2- and 3'UTR-Mut1/2-containing Egfp mRNA cell lines. Enrichment obtained after using the anti-sens specific (Specific Oligonucleotide 2: SO2) or a non-specific (NSO) oligonucleotide probes. ***p<0.001vs non-specific oligonucleotide probe.

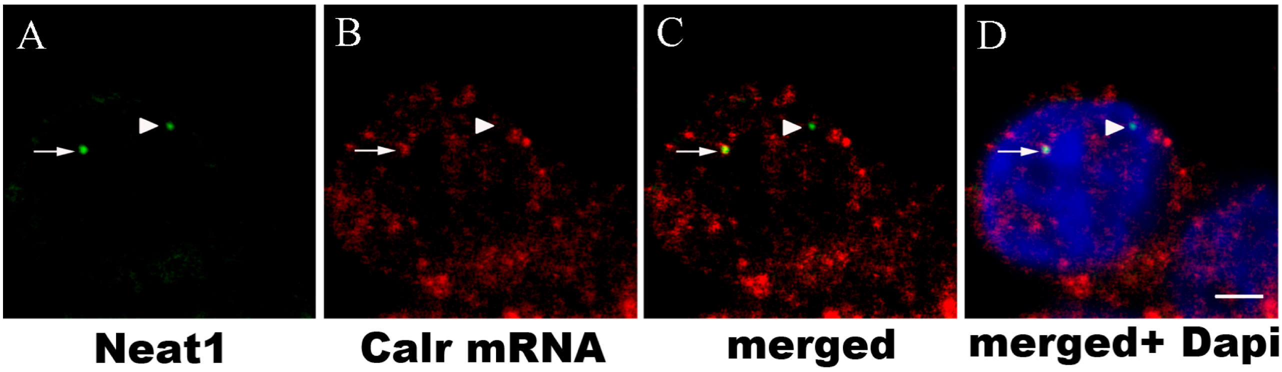

Supplemental Figure S2: Dual FISH visualization of Calr mRNA localizing to paraspeckles

Dual Calr and Neat1 RNA-FISH to pituitary GH4C1 cells show **A.** the nuclear distribution of Neat1 RNA in a few distinct foci (arrow and arrow head). **B.** the cytoplasmic and diffuse nuclear localization of Calr mRNA and its distribution in some nuclear distinct foci. **C.** foci in which Calr and Neat1 RNA overlap (arrow) indicating the paraspeckle localization of Calr mRNA and foci without overlap (arrow head). **D.** Nuclear staining by Hoechst is added to C. Scale bars equal 5 µm.

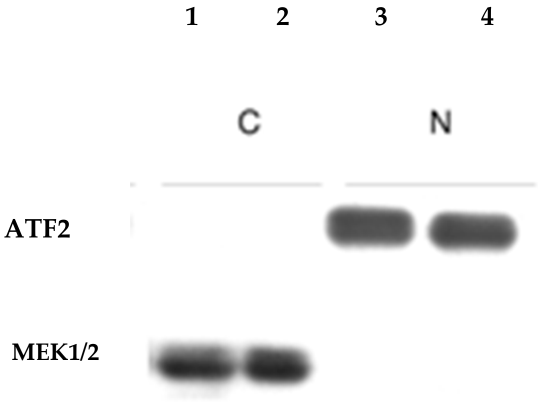

Supplemental Figure S3: Lack of cross contamination between cytoplasmic (lanes 1 and 2) and nuclear (lanes 3 and 4) protein extracts illustrated in two independent samples.

In nuclear and cytoplasmic cell extracts, cross contamination was ruled out by showing that a nuclear transcription factor, activating transcription factor 2 (ATF2) detected using antiATF2 antibodies (1 : 1000 final dilution; Santa Cruz, CA, USA) was expressed exclusively in nuclear fraction whereas a cytoplasmic protein, mitogen-activated protein kinase kinase 1/2 (MEK1/2), detected using anti-MEK1/2 antibodies (1 : 2000 final dilution; Cell Signaling) was expressed exclusively in cytoplasmic fraction.
C: Cytoplasm; N: Nucleus

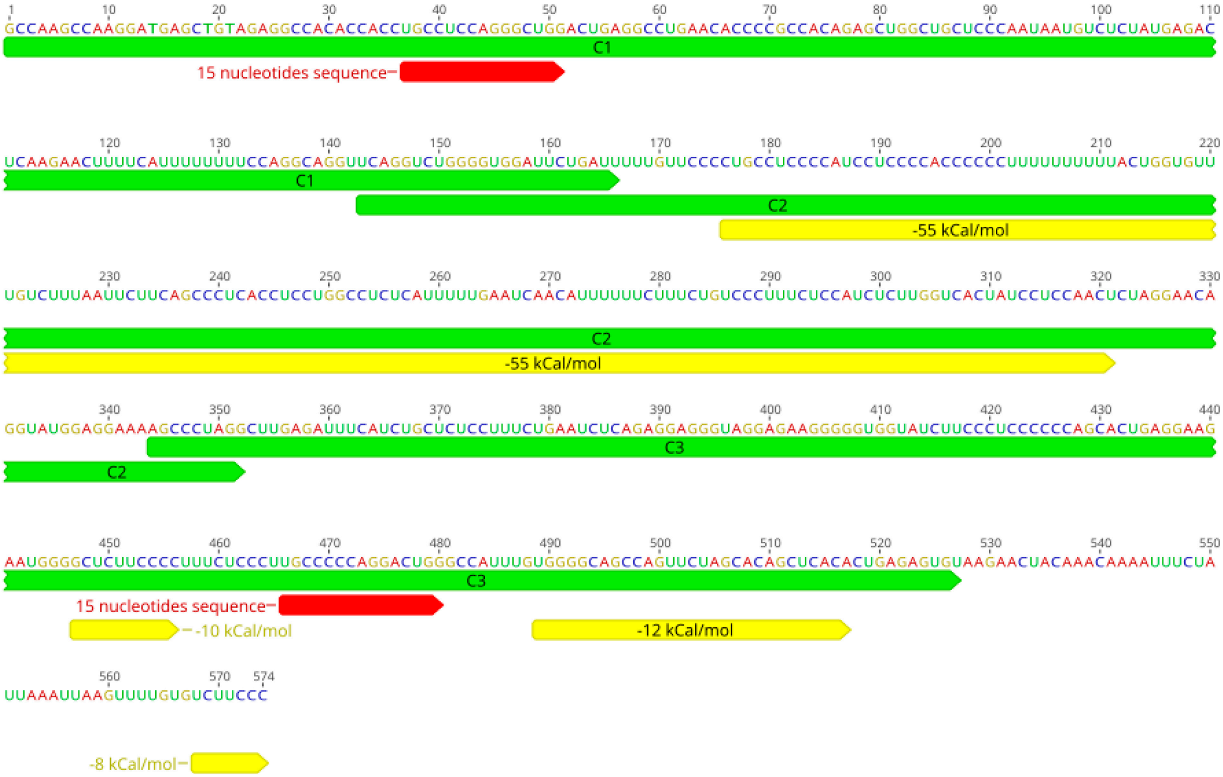

Supplemental Figure S4: Sequence of cloned 3'UTR-Calr with delineation of C1, C2 and C3 fragments (in green).

The 15-nucleotide sequences (in red) found in C1 and C3 are positioned. Regions in 3'UTR exhibiting a significant prediction score for Neat1 interaction are indicated in yellow. The most significant prediction score is found in C2 (-55kCal/mol).

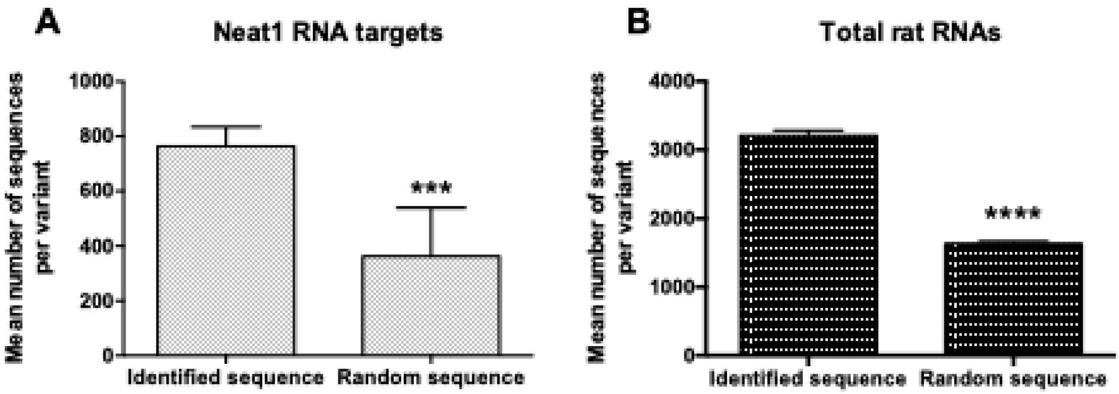

Supplemental Figure S5: Comparison of the number of occurrences of the 15-nucleotide sequence identified here with that of a 15-nucleotide sequence randomly generated, both with Y or R in 5th and 11th position.

A. Mean values for the 4 variants of the 15-nucleotide sequence and for the 4 variants of 30 randomly generated 15-nucleotide sequences found in the 3′UTR of Neat1 RNA targets. **B.** Mean values for the 4 variants of the 15-nucleotide sequence and for the 4 variants of 30 randomly generated 15-nucleotide sequences found in the 3′UTR of all rat RNAs. ***p<0.01 ****p<0.001

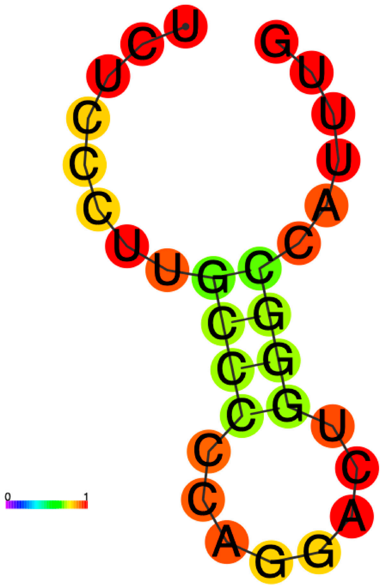

Supplemental Figure S6: Predicted structures of the single stranded 30-nucleotide RNA sequence by RNAfold web server (http://rna.tbi.univie.ac.at/cgi-bin/RNAWebSuite/RNAfold.cgi).

Structure is based on the minimal free energy (MFE) method (an established method to predict RNA structure). Complimentary regions are evaluated to predict the most energetically stable molecule.

Supplemental Table S1: Sequences of qPCR primers and oligonucleotides

qPCR Primer list (rat) (5′ to 3′)

Neat1-1 and 1-2
AAGGCACGAGTTAGCCGCAAAT
TGTGCACAGTCAGACCTGTCATTC

Neat1-2
GCCTGCTTTCAGCTGTTGGTTT
TCTGGACAGCAACTGAGCAATACG

Egfp
CGGCATCAAGGTGAACTTCAAGATCC
ACTGGGTGCTCAGGTAGTGGTT

Calr pre-mRNA
GTGTCCACCTCTGTTCATCTGGTT
AGAGGTCTAAGCCCAGTACAGCAA

Calr
ACCAGAAGGACATGCATGGAGACT
TTGTTGATCAGCACGTTCTTGCCC

Gapdh
CCCTCAAGATTGTCAGCAATG
GTCCTCAGTGTAGCCCAGGAT

Calr 3′ RACE

GSP1: CAGAGAAGCAGATGAAGGACAAGCAG
GSP2: GCCAAGCCAAGGATGAGCTGT

Cloning of 3′UTR Calr fragments

C1: GCCAAGCCAAGGATGAGCTGTAG
AATCAGAATCCACCCCAGACCTGAAC

C2: TCAGGTCTGGGGTGGATTCTGATTT
CCTAGGGCTTTTCCTCCATACCTGT

C3: AGCCCTAGGCTTGAGATTTCATCTGC
ACACTCTCAGTGTGAGCTGTGCTA

Mutagenesis of C1 and C3
$C1_M$:
Forward 5'-GAGGCCACACCACCAGGCACGACGCCAGCACTGAGGCCTGAAC-3'
Reverse 5'- GTTCAGGCCTCAGTGCTGGCGTCGTGCCTGGTGGTGTGGCCTC-3'
$C3_M$:
Forward 5'-GCTCTTCCCCTTTCTCCCTAGGCGCGAGGTCAGCGCCATTTGTGGG-3'
Reverse 5'-CCCACAAATGGCGCTGACCTCGCGCCTAGGGAGAAAGGGGAAGAGC-3'

Neat1 RNA pull-down specific oligonucleotides (3′ biotinylated with a triethyleneglycol spacer)

S oligo 1: CTCCACCATCATCAATCCTCTGGAC
S oligo 2: GCCTTCCCACATTTAAAAACACAAC

Non-specific: ATAATTTCAAACATCAAATGGTATTTTA

RNA protein pull-down specific oligonucleotides (3′ biotinylated with a triethyleneglycol spacer)

30S: U*C*U*C*C*C*UUGCCCCCAGGACUGGGCCAUUUG

30SSM: U*C*U*A*U*A*UUGCCCCCAGGACUGGGCCAUUUG

30NS: U*C*U*A*U*A*UAGGCGCGACGUCAGCGCCAUUUG

*Phosphorothiorate bonds
